# Canopy physiology, vine performance and host-pathogen interaction in a fungi resistant cv. Sangiovese x Bianca accession vs. a susceptible clone

**DOI:** 10.1038/s41598-017-05530-7

**Published:** 2017-07-20

**Authors:** S. Poni, G. Chiari, T. Caffi, F. Bove, S. Tombesi, A. Moncalvo, M. Gatti

**Affiliations:** Department of Sustainable Crop Production, Università Cattolica del Sacro Cuore, Via Emilia Parmense 84, 29122 Piacenza, Italy

## Abstract

The present study compares the physiological and cropping response of the new fungi-resistant grapevine Accession 72–096 (‘Sangiovese’ x ‘Bianca’ hybrid) against a susceptible ‘Sangiovese’ clone which was either fully (FS-SG) or partially sprayed (PS-SG). Data logged on Accession 72–096 indicate that while two early season sprays were enough to avoid major downy mildew (DM) and powdery mildew (PM) outbreaks, Accession 72–096 also showed concurrent desirable features such as moderate cropping, loose clusters, fast sugar accumulation coupled with sufficient acidity even at peak total soluble solids (TSS) concentration (around 24 °Brix), good color and higher flavonols prompting co-pigmentation. Conversely, FS-SG showed final lower acidity despite the notably lower sugar concentration (≅18 °Brix), as well as larger clusters and berries that resulted in more compact bunches. From a methodological viewpoint, end of season single-leaf readings appeared to overestimate the limitation of leaf function due to PM and DM infections in SG-PS vines which, when assessed via a whole-canopy approach, did not show significant differences vs. Accession 72–096, a result likely due to counteracting effects linked to a compensation mechanism by healthy tissues. Our data also suggest that a PM infection can lead to a decoupling in sugar-color accumulation patterns.

## Introduction

The grapevine and its crop are very demanding when it comes to pest and disease management. Controlling the two most insidious diseases, i.e. downy and powdery mildew (*Plasmopara viticola* and *Erysiphe necator*), accounts for about 70% of treatment outlays^1^. In France, for example, 20% of agricultural pesticides are used in vineyards although grapes account for only 3% of the cultivated acreage^2^. Of the 70 M tons of fungicides sprayed in Europe on vines annually, 53.3 M (76%) are used to control powdery mildew (PM) at a cost of €165 M per year^1^.

The front line of these contingent research efforts focuses on pruning and canopy management techniques^3, 4^. The overall aim is to increase indirect tolerance to disease either by improving the microclimate around the fruiting area or by inducing better cluster and/or berry morphology, an example of the latter being looser clusters that are less susceptible to rot diseases due to improved air circulation within the cluster and diminished skin and lesion contact points among adjacent berries. The first, most powerful technique is leaf removal. When applied early in the season, i.e. pre-bloom with flowers still closed, it achieves consistently greater cluster looseness and relative skin growth, making the berry also more tolerant to overheating and sunburn^5, 6^. The second is mechanistic, epidemiological modeling based on disease x weather relationships in the vineyard to provide reliable early warning of approaching outbreak conditions of key diseases, thereby enabling substantial saving of chemical treatments at unchanged protection efficacy^7^. The third combines machinery and intelligent systems: over-row sprayers equipped with a recovery and recycling system of chemical runoff that include variable-rate applications based on geo-referenced real time canopy filling and vigor maps can lead to a remarkable reduction of fungicides^8, 9^. The fourth regards new bio-control agents for powdery mildew. Trials indicate that two applications of Ampelomyces spp. at the peak yellow chasmothecia attack (usually late summer to mid-fall) at 2/3-week intervals effectively reduce the overwintering inoculum and, consequently, disease pressure over the next season^10, 11^.

The last approach is genetics. It is specifically aimed at breeding strategies for introgressing disease-resistant genes in *Vitis vinifera* cultivars that, as is well known, lack native resistant genes to DM, PM and grey mold, *Botrytis cinerea* ^12^. However, DM is a quantitative trait in the genus *Vitis*. For instance, the cv. ‘Bianca’ vine has retained resistance to DM that originally arose in its North American ancestors through several cycles of back-crossing with susceptible cultivars of *Vitis vinifera* followed by genotypic selection^13^. In ‘Bianca’, resistance is controlled by a major dominant gene causing localized necrosis at the infection site. *Vitis rotundifolia* spp. is known to be generally resistant to PM^14^, and one source of resistance conferred by a single dominant locus, called Run1, has been successfully introgressed in V. *vinifera* ^15^. The Run1-mediated defense response involves the induction of a programmed cell death (PCD) mechanism within the penetrated epidermal cells, approximately 12–24 hours following penetration, that arrests fungal development after the formation of a short secondary hypha^16^. A number of accessions of wild Chinese *Vitis* spp. also show significant PM resistance, although the defense response mechanism is still unknown^17^. More recently, Ivanisevic *et al*.^18^ have shown that the introgressed white cv. ‘Pannonia’ shares resistance to both DM and PM.

The ultimate goal of sequential backcrossings with susceptible cultivars of *Vitis vinifera* is to attain a hybrid that retains a minimum of non-*vinifera* blood so that fungi resistance and good enological features can coexist. The process of selection and phenotyping has been greatly accelerated by marker-assisted selection (MAS). MAS application to a number of hybrids developed by the Institute of Applied Genomics (IGA), Udine, Italy, at the end of the 1990s has led to the release of a number of resistant biotypes; registration was completed in 2015 for ten of these new accessions^19^. Susceptible *vinifera* varieties used in this breeding program were ‘Cabernet Sauvignon’, ‘Merlot’, ‘Sauvignon blanc’, ‘Sangiovese’ and ‘Tocai’. Resistance resources were ‘Bianca’ (‘Seyve Villard’ 12–375 x ‘Bouvier’), ‘Regent’ [(‘Sylvaner’ x ‘Muller-Thurgau’) x ‘Chambourcin’], ‘Seyval’ and ‘Pannonia’ (‘Riesling b.’ x SK 86-2/293).

Of the available yet still unregistered accessions, the hybrid ‘Sangiovese’ x ‘Bianca’ (accession 72–096) appears to be the most promising since ‘Sangiovese’ is the most widely grown of Italian red varieties at over than 71,000 hectares^20^ and used in premium labels such like Chianti and Brunello di Montalcino. However, field performance and enological assessment are essentially limited to the work being done by a private company that holds the rights of material storage and propagation^21^. While combining DM and PM resistance with enological attitudes is indispensable, knowledge about leaf and canopy physiology in comparison to the susceptible parental Sangiovese would also be highly valuable. For instance, biometric leaf traits recorded on 72–096 show thinner leaves with much more pronounced main and lateral sinuses. Such characteristics might affect both gas exchange and properties of mass transfer with the surrounding environment (i.e. cooling efficiency).

**Figure 1:**
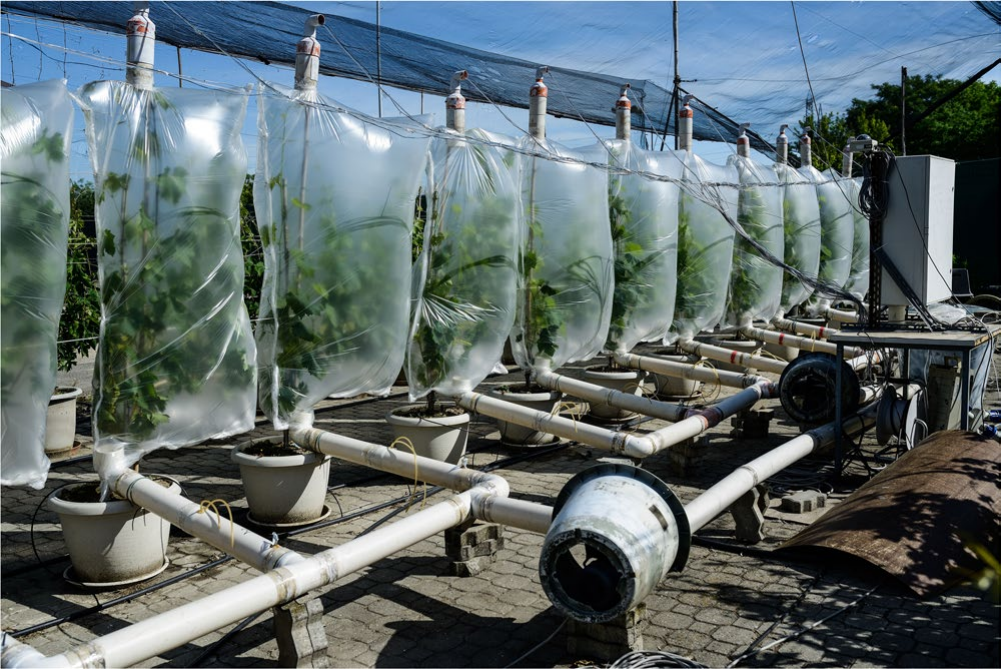
General view of the whole-canopy gas exchange system set up at the beginning of measurements.

The present study combines proximate and ultimate aims. It first compares vine performance, grape composition and single-leaf gas exchange of Accession 72–096 and a susceptible ‘Sangiovese’ (SG) clone either fully or partially sprayed against DM and PM. It then reports seasonal evaluation of whole-canopy photosynthesis (Fig. 1) of Accession 72–096 and partially sprayed SG to establish relationships between disease incidence and severity, carbon assimilation and must composition.

## Results

### Disease incidence and severity

While leaves and clusters of FS-SG had no DM and PM symptoms, PS-SG had 30.8% leaf DM incidence on first assessment, which decreased to 16.7% in late season (Table 1). DM incidence on clusters exhibited an opposite trend: the 27.6% incidence on first date was lower than the 47.2% found at harvest. While PS-Access had no DM on either leaves or clusters on first assessment date, it scored 37.5% incidence on leaves and 7.1% on clusters in late-season estimates. On first date, PM symptoms were noted on PS-SG clusters only (12.7%), whereas on 30 August PM incidence on PS-SG leaves and clusters were 37.5% and 30.06%, respectively. Negligible PM incidence was found on the last date on leaves and clusters of PS-Access (Table 1). Erineum mite had about 50% incidence on PS-Access regardless of assessment date; the same pest was also found on FS-SG and PS-SG leaves, albeit at lower incidence (14–31% range). No erineum mite was spotted on clusters of any treatment.

**Table 1:** Incidence (%) of downy mildew, powdery mildew and erineum mite evaluated during disease assessments on June, 28^1^ and August 30^2^.

	Assessment date	Treatment	Disease Incidence(%)			Total incidence (%)
			Downy mildew	Powdery mildew	Erineum mite	
Leaves	June 28^1^	PS Access	0.00c	0.00c	51.66°	51.66
		PS-SG	30.83a	0.00c	30.83b	61.66
		FS-SG	0.00c	0.00c	24.19b	24.19
	August 30^2^	PS Access	37.50a	7.50b	50.00a	95.00
		PS-SG	16.66b	37.50a	23.33b	77.50
		FS-SG	0.00c	0.00c	14.78c	14.78
Clusters	June 28	PS-Access	0.00c	0.00c	—	0.00
		PS-SG	27.55b	12.66b	—	40.22
		FS-SG	0.00c	0.00c	—	0.00
	August 30	PS-Access	7.10c	2.08c	—	9.19
		PS-SG	47.16a	30.06a	—	77.23
		FS-SG	0.00c	0.00c	—	0.00

Small letters obtained by Fisher protected Least Significant Difference (LSD). Test show significant differences for P < 0.05. PF = partially sprayed. FS = fully sprayed.

^1^BBCH 77 berries beginning to touch; ^2^BBCH 89 berries ripe for harvest; °Brix ≅ 18.

DM severity posted the highest value (6.25%) in late-season scoring on PS- SG clusters (Table 2); the highest PM severity was reached in late season PS-SG leaf assessment (19%); all remaining treatment x date combinations never exceeded 2% severity. Erineum mite severity mirrored incidence data: the highest values of 15% and 12% were reached in PS-Access for mid-season and late season assessment, respectively.

**Table 2:** Severity (%) of downy mildew, powdery mildew and erineum mite evaluated during disease assessments on June, 28^1^ and August, 30^2^.

	Assessment date	Treatment	Disease Severity (%)			Overall Severity (%)
			Downy mildew	Powdery mildew	Erineum mite	
Leaves	June 28^1^	PS Access	0.00c	0.00b	15.00a	15.00
		PS-SG	2.90a	0.00b	2.40b	5.30
		FS-SG	0.00c	0.00b	1.65b	0.00
	August 30^2^	PS Access	3.70a	1.40b	12.00a	17.10
		PS-SG	1.80b	19.00a	3.70b	24.50
		FS-SG	0.00c	0.00b	2.07b	0.00
Clusters	June 28	PS-Access	0.00c	0.00c	—	0.00
		PS-SG	3.08b	0.32b	—	3.39
		FS-SG	0.00c	0.00c	—	0.00
	August 30	PS-Access	0.18c	0.05bc	—	0.23
		PS-SG	6.25a	1.76a	—	8.01
		FS-SG	0.00c	0.00c	—	0.00

Small letters obtained by Fisher protected Least Significant Difference (LSD). Test show significant differences for P < 0.05. PF = partially sprayed. FS = fully sprayed.

^1^BBCH 77 berries beginning to touch; ^2^BBCH 89 berries ripe for harvest; °Brix  ≅ 18.

### Single-leaf and whole-canopy gas exchange

PS-Access usually had higher leaf assimilation rates (A) than SG, whether FS or PS (Table 3). However, such tendency did never reach significance when compared to FS-SG rates, whereas A rates of PS-Access were significantly higher (P < 0.05) than those measured on PS-SG for readings taken on DOYs 173 and 244. Conversely, leaf water status expressed as both transpiration (E) and stomatal conductance (g_s_) was much less responsive than A (Table 3) and correlations between g_s_ and A and between E and A for data pooled over the three treatment levels resulted to be non significant (R^2^ < 0.15) on both sampling dates; consequently, water use efficiency (WUE), regardless of expression, reflected relative changes of A, E and g_s_. WUE_inst_ was considerably lower in PS-SG on final date, while WUE_i_ was significantly reduced in the same treatment at both DOYs 173 and 244.

**Table 3:** Leaf assimilation (A), transpiration (E) and stomatal conductance (g_s_) measured on single leaves of SG (susceptible clone R10) and accession 72-096 (resistant)

Treatment	A (μmol m^−2^s^−^1)			E (mmol m^−2^s^−1^)			g_s_ (mmol m^−2^s^−1^)			WUE_inst_ A E^−1^			WUE_i_ A g_s_^−1^		
DOY	128	173	244	128	173	244	128	173	244	128	173	244	128	173	244
PS-SG	13.3	10.9b	3.6b	5.84	5.92	5.42	231b	283	189	2.31	1.84	0.68b	57.8	38.5b	19.5b
FS-SG	14.2	11.9ab	6.5a	5.99-	5.86	5.18	250ab	273	178	2.37	2.03	1.25a	56.8-	43.5ab	36.5a
PS-Access	15.1	14.7a	6.8a	6.31	6.42	5.56	271a	278	215	2.39	2.29	1.24a	56	52.8a	31.6a
Sig.	ns	*	*	ns	ns	ns	*	ns	ns	ns	ns	*	ns	**	*

prior whole canopy system setup (May 18, DOY 128) and right after first and final dismantling (22 June, DOY 173 and 1 September, DOY 244, respectively). Instantaneous water use efficiency (WUE_inst_) and intrinsic (WUE_i_) were calculated accordingly. PF = partially sprayed. FS = fully sprayed.

* and ** denote significant differences between treatments at P < 0.05 and 0.01 according to within column mean separation performed with SNK test and showed by small letters. ns = not significant.

**Figure 2:**
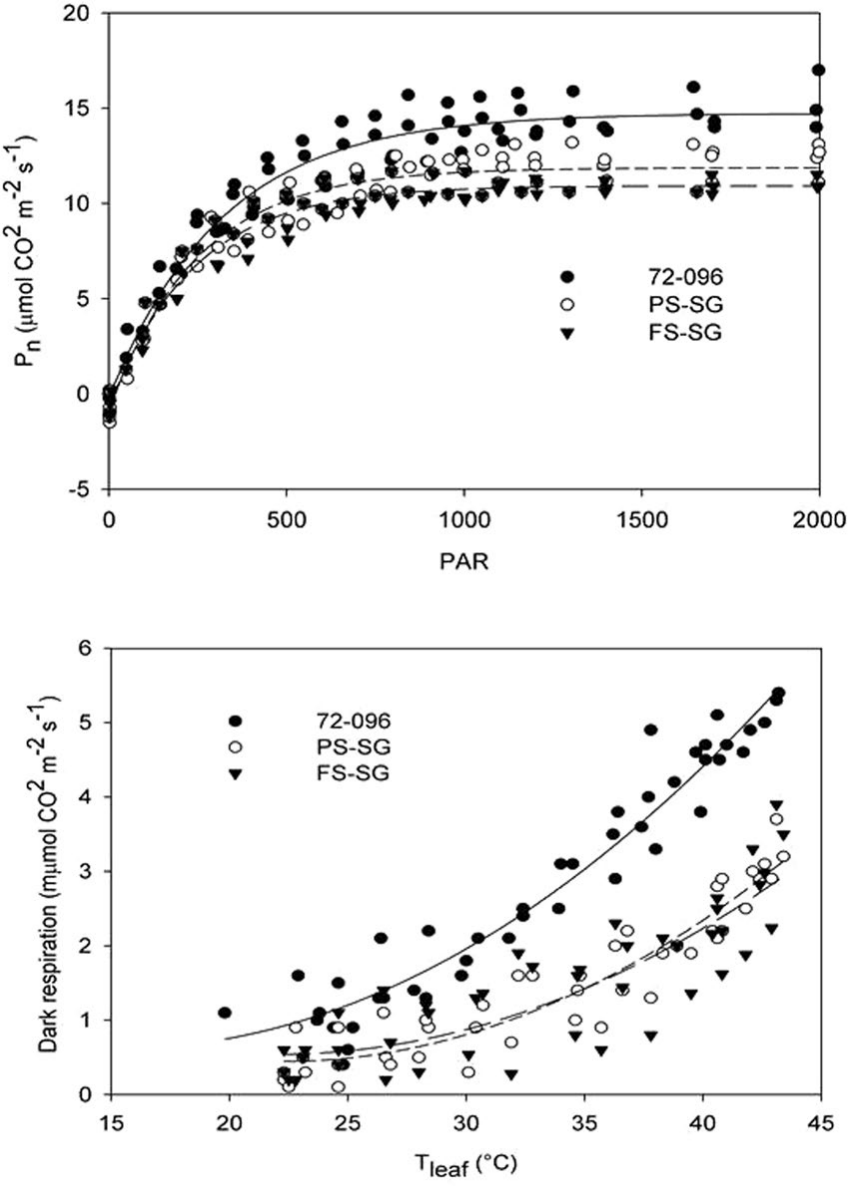
(**a**) Leaf net photosynthesis (P_n_) vs. light intensity (PAR) of fully expanded leaves of Accession 72-096 (y = −0.1908 + 14.9208*(1 − e^(−0.0032x)^), R^2^ = 0.94, P < 0.001), FS-SG (y = −0.6954 + 11.6205*(1 − e^(−0.0042x)^), R^2^ = 0.95, P < 0.001) and PS-SG (y = −0.8528 + 12.7258*(1 − e^(−0.0040x)^), R^2^ = 0.94 P < 0.001); (**b**) Leaf dark respiration (R_D_) vs. leaf temperature (T_leaf_) of fully expanded leaves of 72-096 (y = 2.1066 − 0.1924x + 0.0063x^2^ R^2^ = 0.94 P < 0.001), FS-SG (y = 3.0714 − 0.2300x + 0.0052x^2^ R^2^ = 0.87, P < 0.001) and PS-SG (y = 3.4158 − 0.2674x + 0.0060x^2^ R^2^ = 0.73; P < 0.001). Measurements were carried out between 20 and 24 June 2016 on 5 mature leaves per treatment.

Light response curves drawn on DOY 173, while showing no substantial difference in light saturation and compensation points between treatments, confirmed that A rates recorded in PS-Access were generally higher than those measured in SG at saturation and beyond (Fig. 2a). Interestingly, leaf dark respiration (R_D_) vs. leaf temperature (T_leaf_) plots showed that R_D_ was consistently higher in PS-Access at any T_leaf_ level (Fig. 2b).

**Figure 3:**
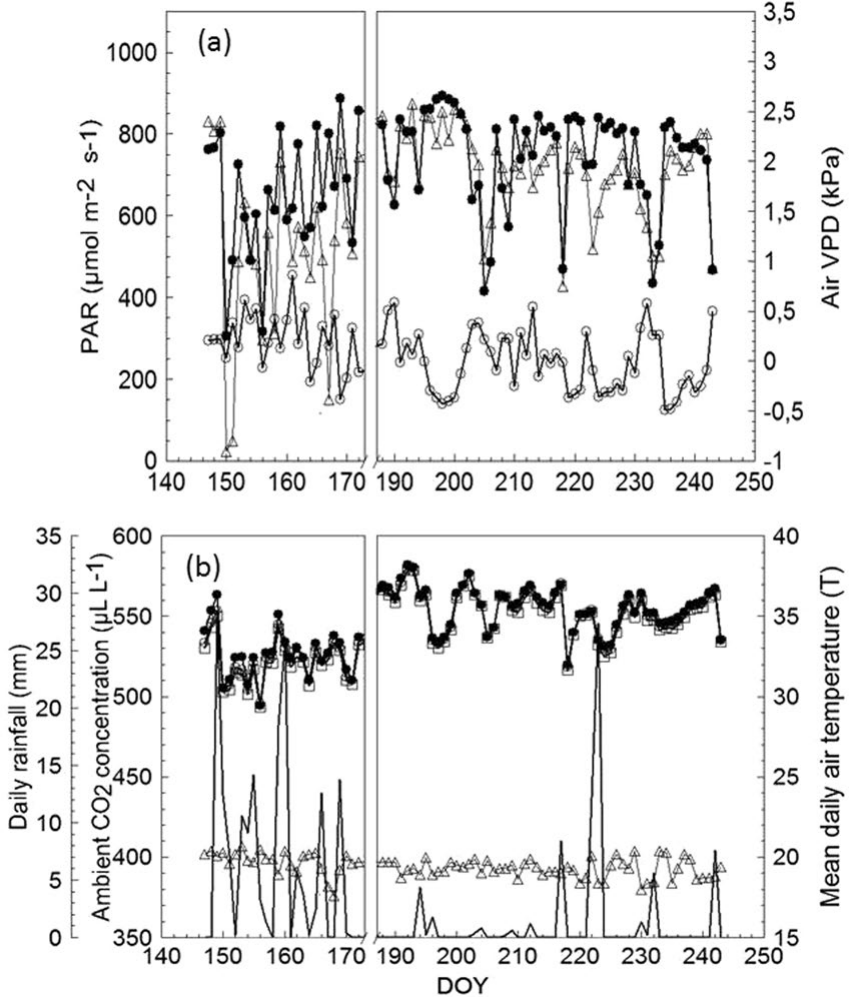
(**a**) Seasonal trends of direct (●) and diffuse (○) photosynthetically active radiation (PAR) and air vapor pressure deficit (VPD); (**b**) inlet chamber air temperature (◻), outlet chambers air temperature for PS-SG (●), outlet chambers air temperature for Accession 72-096 (○ and ambient CO_2_ concentration (▵) measured at the trial site. Values are daily means averaged from dawn to dusk.

The first cycle of whole-canopy gas exchange measurements (DOY 147–172) was marked by quite variable weather with abundant rainfall (Fig. 3). Air-to-leaf VPD slightly exceeded 2 kPa at the beginning of the period; mean diurnal air temperature peaked at 30 °C on DOY 150 and then fluctuated around 25 °C over the remaining days. The second period of measurement (DOYs 188–243) was definitely warmer with a high frequency of clear days: air-to-leaf VPD peaked at 2.5 kPa and mean diurnal air temperature often exceeded 30 °C.

**Figure 4:**
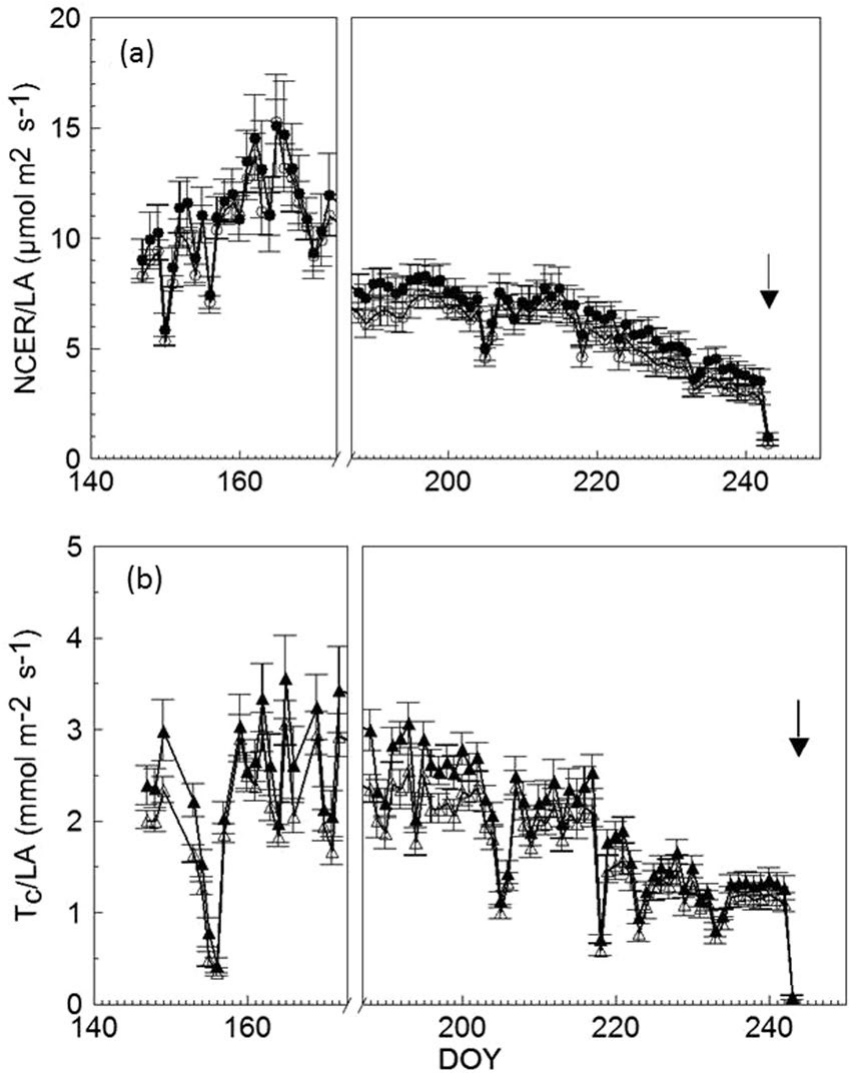
(**a**) Seasonal trends of daily mean net CO_2_ exchange rate per unit leaf area (NCER/LA) measured in 72-096 (●) and PS-SG (○) and (**b**) seasonal trends of whole-canopy transpiration per unit leaf area (T_c_/LA) measured on 72-096 (▴) and PS-SG (▵). Vertical bars indicate standard error (n = 6). Arrow indicates harvest date.

Net CO_2_ exchange rate (NCER) normalized vs. total leaf area (LA) per vine (µmol m^−2^ s^−1^) averaged over the whole first measuring period was 11.1 ± 0.43 µmol m^−2^ s^−1^ (mean ± SE) in PS-Access vs. 10.5 ± 0.42 µmol m^−2^ s^−1^ recorded in PS-SG (Fig. 4a). Similar non-significant differences were recorded over the second whole measuring cycle with PS-Access scoring an NCER of 6.2 ± 0.63 µmol m^−2^ s^−1^ as compared to 5.4 ± 0.52 µmol m^−2^ s^−1^ in PS-SG. The NCER response was essentially mirrored by canopy transpiration (T_C_) per unit of leaf area (Fig. 4b). Averaged over the first measuring period, mean T_C_ in PS-Access was 2.39 ± 0.18 mmol m^−2^ s^−1^ in 72–096 and 2.07 ± 0.17 mmol m^−2^ s^−1^ in PS-SG. Mean T_C_ of PS-Access over the second measuring cycle was 1.87 ± 0.14 mmol m^−2^ s^−1^ as compared to 1.63 ± 0.14 mmol m^−2^ s^−1^ in PS-SG. As a result of NCER and T_C_ trends, canopy WUE, calculated as NCER to T_C_^−1^ ratio, was clearly unaffected by treatments, showing 4.64 ± 0.81 mmolCO_2_ molH_2_0^−1^ and 5.08 ± 1.16 mmolCO_2_ molH_2_0^−1^ for 72–096 and PS-SG, respectively, for the first period, and 3.57 ± 0.54 and 3.60 ± 0.32 mmolCO_2_ molH_2_0^−1^, in that order, for the second period.

### Yield components and must composition

Shoot number was very similar across treatments (Table 4). Yield per vine recorded at harvest was slightly lower in PS-Access (2.9 kg) as compared to SG, as both FS and PS posted about 3.4 kg (Table 4). A look at yield components shows that PS-Access scored higher shoot fertility and, hence, higher cluster number than PS-SG. By contrast, cluster and berry weight, as well as berries per cluster, were lowest in PS-Access; it also showed lowest cluster thickness compared to the other treatments despite a shorter rachis length. The source-to-sink balance given as leaf area-to-fruit ratio was similar and slightly lower than 1 m^2^ kg^−1^ of fruit fresh mass. Comparison within the SGs showed that a different level of protection resulted in smaller clusters in PF-SG, a consequence primarily due to a significantly reduced berry number per cluster (Table 4).

**Table 4:** Vegetative growth, yield components and vine balance recorded on vines of SG (susceptible clone R10) and accession 72-096 (resistant). PF = partially sprayed. FS = fully sprayed.

	Shoots/ vine	Clusters/vine	Cluster weight (g)	Berry weight (g)	Berries/ cluster	Rachis length (cm)	Cluster thickness (g cm^−1^)	Yield/ vine (kg)	Leaf area/vine (m^2^)	LA/yield ratio (m^2^ kg^−1^)
PS-SG	8.0	13.3ab	257b	2.775a	113b	14.8	19.0a	3.413	2.9032	0.854
FS-SG	7.0	11.2b	303a	2.692a	131a	16.1	21.8a	3.402	2.5890	0.761
PS-Access	8.3	15.8a	184c	2.080b	99b	11.7	15.7b	2.915	2.9145	0.995
Significance	ns	*	**	**	ns	**	*	ns	ns	ns

Cluster thickness is given as total fresh berry mass to rachis length ratio (g/cm). LA = leaf area

* and ** denote significant differences between treatments at P < 0.05 and 0.01 according to within column mean separation performed with SNK test and showed by small letters. ns = not significant.

**Figure 5:**
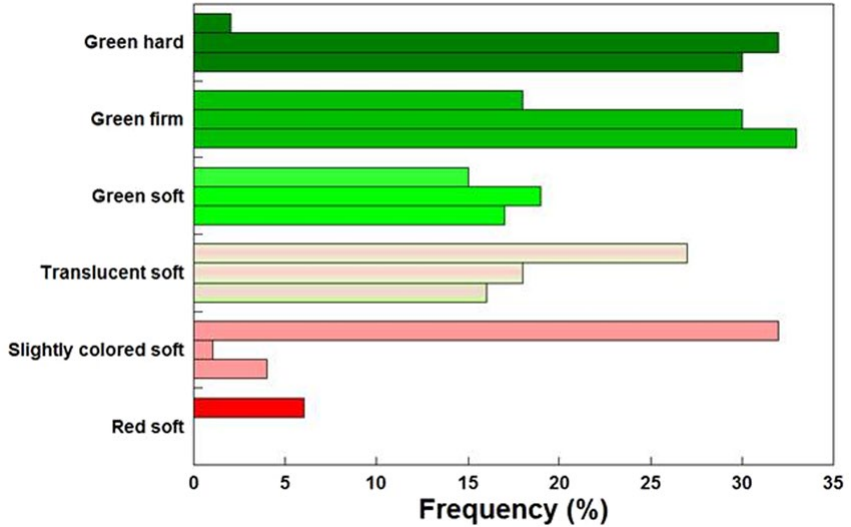
Frequency distribution as per six different berry ripening categories recorded on 14 July on a 100-berry sample per treatment. Within each category, top bar is PS-Access, mid is PS-SG and bottom is FS-SG. Mean soluble solids concentration (°Brix) of each 100-berry sample was 8.64, 6.54 and 6.31 for 72-096, PS- SG and FS-SG, respectively.

The onset of veraison observed on a 100-berry sample per treatment at 14 July (DOY 195) showed clear advancement in PS-Access: its 32% maximum frequency category was represented by ‘slightly colored soft berries’; ‘green hard’ and ‘green firm’ berries were the categories most represented in PS- and FS-SG, respectively (Fig. 5). Mean TSS determined on the 100-berry samples (grand mean of individual berries) was 8.6 °Brix in PS-Access against 6.5 and 6.3°Brix, respectively, in PS- and FS-SG.

Must composition was notably affected by treatment. PS-Access reached a remarkable 24.3°Brix TSS at harvest, whereas both SGs were around 18°Brix (Table 5). While must pH was unchanged, titratable acidity differed, scoring 6.8 g L^−1^ in PS-Access and <5.5 g L^−1^ in the two SGs. Total anthocyanin concentration (mg g^−1^) and profile of single anthocyanins, either mono or di-glucoside, also differed (Tables 5 and 6). The former was highest in FS-SG at 13.95 mg g^−1^ (skin dry weight), intermediate in PS-Access at 10.81 mg g^−1^ and lowest in PS-SG at 5.62 mg g^−1^. These differences were supported by the mono-glucoside anthocyanin count expressed as the summation of Dp + Pt + Mv and Cy + Pn; acetyl and coumaroyl derivatives were more abundant in PS-Access (Table 5). As expected, di-glucoside forms were not detected in *vinifera* SG, whereas they were present in PS-Access. The anthocyanin profiles in Table 6 show that Mv3-O-glc was the most abundant at 30.6% and 40.6% of the total anthocyanin pool in PS- and FS-SG, respectively, whereas Cy3-O-glc was the prevalent form in PS-Access at 24.4%.

**Table 5:** Must composition recorded at harvest on SG (susceptible clone R10) and accession 72-096 (resistant). PF = partially sprayed. FS = fully sprayed. Anthocyanins data expressed as mg of malvidin-3-O-glucoside equivalent per g of skin dry weight. Abbreviations: Anth: anthocyanins, Dp: delphinidin, Cy: cyanidin, Pt: petunidin, Pn: peonidin, Mv: malvidin, acyl: acylated, coum: coumarated, glc: glucoside, n.d.: not detected.

	TSS (Brix)	pH	TA (g L^−1^)	Total sugars/ vine (g)	Total Anthoc.(mg/g)	Dp + Pt + Mv 3,5-O-glc (mg/g)	Cy + Pn 3,5-O-glc (mg/g)	Dp + Pt + Mv 3-O-glc (mg/g)	Cy + Pn 3-O-glc (mg/g)	Anth. acyl. (mg/g)	Anth. coum. (mg/g)
PS-SG	18.3b	3.41	4.86b	62.4	5.62c	n.d.	n.d.	2.81b	2.72b	0.02b	0.07b
FS-SG	17.9b	3.40	5.41b	60.9	13.95a	n.d.	n.d.	7.97a	5.85a	0.03b	0.10b
PS-Access	24.3a	3.41	6.81a	70.6	10.81b	1.28	1.91	3.84b	3.54b	0.05a	0.19a
Sig.	**	ns	**	ns	***	—	—	***	***	**	***

** and *** denote significant differences between treatments at P < 0.01 and 0.001 according to within column mean separation performed with SNK test and showed by small letters. ns = not significant.

**Table 6:** Anthocyanin profile at harvest in berry skins of SG (susceptible clone R10) and accession 72-096 (resistant). PF = partially sprayed. FS = fully sprayed. Data expressed as mg of malvidin-3-O-glucoside equivalent per g of skin dry weight. Abbreviations: Dp: delphinidin, Cy: cyanidin, Pt: petunidin, Pn: peonidin, Mv: malvidin, glc: glucoside, n.d.: not detected.

	Dp 3,5-O-glc (mg g^−1^)	Cy 3,5-O-glc (mg g^−1^)	Pt 3,5-O-glc (mg g^−1^)	Pn 3,5-O-glc (mg g^−1^)	Mv 3,5-O-glc (mg g^−1^)	Dp-3-glu (mg g^−1^)	Cy-3-glu (mg g^−1^)	Pt-3-glu (mg g^−1^)	Pn-3-glu (mg g^−1^)	Mv-3-glu (mg g^−1^)
PS-SG	n.d.	n.d.	n.d.	n.d.	n.d.	0.47c	1.44b	0.63b	1.28b	1.71b
FS-SG	n.d.	n.d.	n.d.	n.d.	n.d.	0.80b	2.52a	1.50a	3.33a	5.67a
PS-Access	0.14	0.84	0.28	1.07	0.86	1.21a	2.64a	1.21a	0.90b	1.42b
Sig.	—	—	—	—	—	**	**	**	***	***

** and *** denote significant differences between treatments at P < 0.01 and 0.001 according to within column mean separation performed with SNK test and showed by small letters. ns = not significant.

Flavonol composition of berry skins at harvest was unaffected by treatment for myricetin, quercetin glucuronide and kaempferol; the most abundant component was quercetin3-O-glucoside and had the highest value in PS-Access (Table 7). Seed tannin composition was also affected by treatments (−);-epicatechin was especially high in FS-SG along with syringic acid, vanillin and procyanidin B2 (Table 8). Conversely, protocatechuic acid was highest in PS-Access.

**Table 7:** Flavonols composition at harvest of berry skins of SG (susceptible clone R10) and accession 72-096 (resistant). PF = partially sprayed. FS = fully sprayed. Data are given as mg/kg of skin dry weight. Abbreviations: Myr: myricetin, Quer: quercetin, Kmp: kaempferol, gluc: glucuronide, glc: glucoside.

	Myr 3-O-glc (mg kg^−1^)	Quer 3-O-glc (mg kg^−1^)	Quer 3-O-glc (mg kg^−1^)	Kmp 3-O-glc (mg kg^−1)^
PS-SG	116.92	641.53	1373.33ab	37.95
FS-SG	126.05	602.57	1121.53b	24.59
PS-Access	101.45	772.29	1774.68a	31.78
Sig.	ns	ns	*	ns

*Denote significant differences between treatments at P < 0.05 according to within column mean separation performed with SNK test and showed by small letters. ns = not significant.

**Table 8:** Phenolic compounds composition at harvest in seeds of SG (susceptible clone R10) and accession 72-096 (resistant). PF = partially sprayed. FS = fully sprayed. Data expressed as mg kg^−1^ of seed dry weight.

	Gallic acid (mg kg^−1^)	Protocatechuic acid (mg kg^−1^)	Syringic acid (mg kg^−1^)	Vanillin (mg kg^−1^)	(+)-catechin (mg kg^−1^)	(-)-epicatechin (mg kg^−1^)	Procyanidin B2 (mg kg^−1^)
PS-SG	76.60	13.40b	29.45b	27.58b	826.66	801.70b	154.93b
FS-SG	87.20	15.52b	59.01a	57.40a	1185.72	2047.24a	206.38a
PS-Access	79.37	24.43a	25.23b	28.75b	899.11	621.66b	130.81b
Sig.	ns	***	***	***	ns	***	*

* and *** denote significant differences between treatments at P < 0.05 and 0.001 according to within column mean separation performed with SNK test and showed by small letters. ns = not significant.

Principal component analysis carried out on 14 variables to include total vine LA, yield components and main compositional traits showed that cumulated eingenvalues over the first five filter factors accounted for by 89.5% of total variability (Fig. S1), whereas the first two filter factors (F1 and F2) represented, respectively, 39.0% and 21.1% of total variability. The correlation matrix between variable and factors (Table S1) was screened to highlight positive or negative correlation above 0.6. F1 had 8 correlations above thresholds, whereas this number decreased to 3,2,1,1 for the F2, F3, F4 and F5, respectively. Biplot where F1 and F2 axes accounted for by 60.22% of total variance clearly isolated performance of PS-Access (Fig. S2); conversely, position of the two differently sprayed SG overall mixed and no characterizing variables could be identified. F1 strongly characterized the behavior of PS-Access that merged features of high TSS, TA and LA/Y ratio.

## Discussion

Comparing vine performance of PS-Access to that of both SGs makes it possible to discriminate responses in the absence of major disease outbreaks (for PS-Access and FS-SG) and under the same two-spray protection schedule in order to study host-pathogen interaction and its effect on gas exchange and overall vine performance for PS-Access and PS-SG. Our discussion will thus focus on these two data sets.

Given the lack of previous exhaustive data, our yield records emphasize that PS-Access, despite its highest shoot fruitfulness, is a moderate cropping biotype compared to FS-SG, a result of the lower berry set and much smaller berries that notably reduced cluster weight (Table 4) as also nicely confirmed by the PCA biplot showing F1 and F2 (Fig. S2). This profile is very appealing for two main management reasons. First, ‘Sangiovese’ is quite well known as a very fruitful, highly productive cultivar with a pronounced tendency to over-crop that impairs fruit quality. In effect, it is quite responsive to techniques like cluster thinning and pre-bloom basal leaf removal for balancing fruit load^5^. Second, the lower-yielding PS-Access is conducive to improved grape quality. As shown in Table 4, it has looser clusters than FS-SG, a factor that can induce improved air circulation and illumination of internal berries and lower susceptibility to bunch rot. Its lower yield leads to a leaf area-to-yield ratio close to 1 m^2^ kg^−1^, which represents a general threshold below which carbon supply is supposed to become limiting for sugar accumulation^22^. A possible objection to the previous assumption is that LA is given as “total” leaf area that, according to exposure, age, health status, and so forth, can correspond to quite different levels of “functional” leaf area as shown in other work^23, 24^. However, if leaf function can be conveniently expressed by leaf assimilation (A) rate, the data shown in Table 1 demonstrate that at no time during the season did maximum A significantly differ between PS-Access and FS-SG, thereby suggesting that the ratio of functional-to-total LA in these two treatments was likely comparable.

Anthocyanin composition, as expected, showed presence of di-glucoside forms in PS-Access containing non-*vinifera* blood^25^. Anthocyanidin 3,5-O-diglucosides were reported as less stable to oxidation and heat compared to the corresponding mono-glucosides^26^; this may result in rapid color loss during juice storage and wine aging^27^. Moreover, the prevalent anthocyanin in PS-Access is cyanidin 3-O-glucoside (24.4%), a rather unstable anthocyanin glucoside as compared to malvidin 3-O-glucoside that accounts for 40.6% of total anthocyanins in FS-SG. In fact, that malvidin 3-O-glucoside and peonidin 3-O-glucoside do not possess ortho-positioned hydroxyl groups makes them comparatively more resistant to oxidation than cyanidin 3-O-glucoside during barrel aging of red wines^28^.

**Figure 6:**
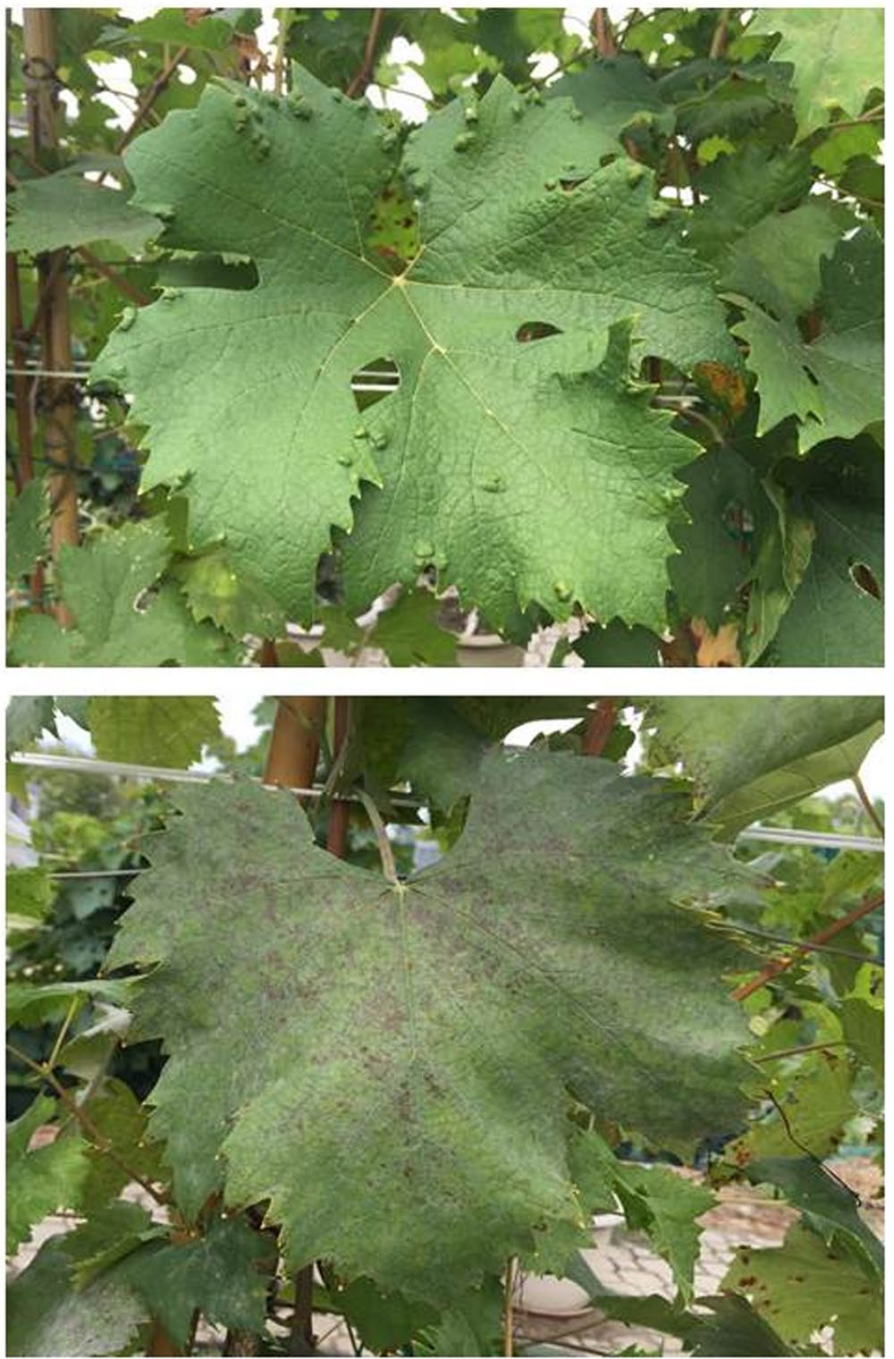
Morphology of a mature leaf of PS-Access (top) and PS-SG (bottom).

However, quercetine 3-O-glucoside, the most abundant flavonol compound, was significantly increased in PS-Access. It has been demonstrated that induction of flavonol synthesis in the grape berry is positively correlated to the amount of exposure to direct light since these components act as sunscreens against UV radiation^29^. Since canopy density and management were uniform across treatments, and that leaf removal was never differentially applied during the season, there seems to be no reason to justify different cluster or berry exposure between the genotypes in our trial. However, as shown in Fig. 6, SG and PS-Access differ quite notably in terms of leaf morphology: SG shows a typical five-lobed leaf with a wide petiolar sinus and no apparent lateral sinuses; PS-Access too has a five-lobe leaf but with a narrow petiolar sinus and quite deep lateral sinuses forming ‘holes’ in the leaf. Thus, the latter morphology would allow more light penetration inside the canopy to clusters and subsequent increase in quercetin synthesis. From an enological standpoint, it is well known that co-pigmentation, i.e. the association between pigments and other, usually uncolored, organic molecules in solution, allows pigments to exhibit far greater color than would be expected from their own concentration^30^. A look at PS-Access’s berry composition shows that quercetin and malvidin combine for a purplish blue hue in an otherwise red solution, and that protocatechuic acid and cyanidin 3,5-O-diglucoside lead primarily to color enhancement^31^. This would support the assumption that the color of young wines produced with grapes including some non-*vinifera* blood can be more intense and charming that that of wines entirely made with *vinifera* ^30^.

Albeit limited to one season and without additional information from micro-vinification of final wines, overall ripening dynamics and must composition shown by FS-SG and PS-Access are so distinctly different as to suggest several marketing features of the final product. Traits shown by PS-Access hint at moderately yielding, well balanced, loose clusters of small berry size that, when compared to the benchmark ‘Sangiovese’ clone, evinced earlier ripening (about one week as estimated at veraison), a fast rate of sugar accumulation coupled, most importantly, with maintenance of sufficient acidity, a quite vivid and crisp color assured by the presence of di-glucoside anthocyanins and higher quercetin, which in turn can promote co-pigmentation. The overall picture is of a quite fresh, easy drinking, fresh colored red wine unsuitable for aging and, hence, might meet current tendencies of wine consumers^3^. The high alcohol content, which may derive from fast sugar accumulation can be easily negotiated by anticipating harvest with a further gain of retained total acidity. Coupled to the potential of targeting a range of new consumers, it is notable too that PS-Access may be well perceived socially since it is managed under a much reduced pesticide regime (2 against the 12 FS-SG sprays). That erineum mite incidence and severity were highest in PS-Access, and some mild DM symptoms were seen on leaves and clusters, should not be taken as major drawbacks. Their appearance is not surprising since durability of resistance depends on the type of resistance itself: a pyramiding strategy is more durable than the use of single resistance genes^32^ and combining resistance with such other means as pesticides, biocontrol, beneficial organisms are examples. Although in our trial we applied only two fungicide treatments in order to safeguard durability and avoid major late-season infection, this schedule does not assure complete protection.

A second major issue relates to disease vs. leaf function relationships of PS-SG and PS-Access, i.e. susceptible against tolerant genotype subjected to the same protection treatment. As expected, reduced pest protection of the former caused DM to develop on leaves and clusters, reaching a 30.8% incidence on leaves by 28 June (DOY 179) with 2.9% severity. This infection rate did not seem to impair single-leaf gas exchange since A rates measured on the closest date (DOY 173) were similar between the SGs. This is not surprising as previous work by Jermini *et al*.^33^ has shown that leaf DM severity below 5% (middle value of the modified Horsfall scale) is unable to modify gas exchange and water-use efficiency in cv. ‘Merlot’. Moreover, the same research group^34^ concluded that if leaf DM severity ranges only from 1% at the onset of veraison to 5% in late August, corresponding in their conditions to the end of the linear phase of rapid soluble sugar accumulation, no significant change in total soluble solids is expected at harvest, as actually occurred in our study when the SGs were compared.

Interestingly, the lower DM incidence and severity we found on leaves at harvest, coupled with a significant reduction of the assimilation rate in PS- vs FS-SG, clearly suggests that the interaction with the concurrent PM spread (37.5% and 19% for incidence and severity, respectively) played a role. However, the relationship between PM levels of leaf infection and leaf function is controversial. Lakso *et al*.^35^ reported that the same level of infected single-leaf area (50%) induced about 50% and 8% reduction in leaf A in cvs. White Riesling and Concord, respectively, suggesting that the response is highly genotype dependent. Our data seem to support this assumption: a 19% PM disease severity (Table 2) caused a 45% reduction in A rates. Conversely, there is a very close match between our and Lakso *et al*.’s work: both corroborate that leaf water status (i.e. E and g_s_ rates) at any PM severity rate was not reduced vis-à-vis healthy tissue and, as a consequence, water-use efficiency decreased regardless of its form of expression (Table 3). Indeed lack of significant correlation between E and g_s_ vs. A rates on DOY 173 and 244 suggest that, especially in PS-SG, the notable A decrease was primarily caused by non-stomatal limiting factors. There is no clue to explain the higher incidence and severity of Erineum mite on leaves of PS-Access as compared to the ‘Sangiovese’ biotypes, which, at least in theory, could be related to different leaf morphology (Fig. 6). Though, presence of Erineum mite on leaves at the observed levels does not seem to affect leaf function since A rates taken at harvest on either symptomless and symptomatic leaves and pooled over the three treatments resulted in 5.15 and 5.41 µmol m^−2^ s^−1^.

Conclusions drawn in the present paper on the effects of PM and DM on single-leaf gas exchange will need to be partially revisited when the comparison is carried out on a whole-canopy basis. While we are not aware of any specific work on grapevine where the effects of DM and PM infections have been monitored on a seasonal, whole-canopy basis, lack of difference in mean NCER between Access and PS-SG recorded over the two measuring periods in Fig. 4a do not match the A rates in Table 3, indicating that PS-Access had higher leaf photosynthesis than PS-SG at DOY 173 and 244. Conversely, lack of seasonal differences in T_c_ between the two treatments (Fig. 4b) is quite a close match of the single-leaf readings. Responses evaluated at both single-leaf and whole-canopy levels therefore raise the interesting issue of which is the ‘true’ effect on vine function and, depending on the method used for disease assessment, which conclusions are actually to be drawn. Direct whole-canopy evaluation overcomes the typical bias of punctual single-leaf readings, which need to be extrapolated to the canopy level with some unavoidable oversimplifications^36^. The advantage of the whole-canopy approach is that it integrates into a single-vine reading a series of complex interacting factors (leaf age, exposure, health, nutritional status, etc.) that even a broad-based single-leaf sampling can barely account for. This seems especially true for determinations related to disease assessment for which previous work has shown that high variability may also occur within the same leaf depending on the leaf portion sampled, i.e. healthy tissue of diseased leaves against infected tissue of diseased leaves^37, 38^. It seems that the lower A rates recorded on PS-SG at DOY 244 for leaves scoring 2.56 disease severity on a 0 to 5 scale (i.e. medium damage) were not low enough to compromise whole NCER, which over the second measuring period was on average only 0.8 µmol m^−2^s^−1^ less than the NCER rate measured on PS-Access (Fig. 4a). This response links to the well-known ability of grapevine leaves to carry high photosynthetic compensation any time a significant source limitation occurs^39^. This mechanism may have been active in the green tissue of healthy and infected leaves on PS-SG vines. In support of the compensation effect, Jermini *et al*. (2010c)^40^ reported that, in a mature ‘Merlot’ grapevine subjected to either untreated or reduced fungicide schedule vs. standard spraying, the significant canopy damage caused by DM in the first two treatments induced a strong remobilization of starch reserves from the woody parts and, especially, from the roots.

Further discussion is also needed about the effects of disease incidence and severity on yield and grape composition. No significant difference in yield per vine between treatments (Table 4) and especially for the comparison within the SG genotype is a quite close fit with the slight seasonal NCER differences detected by the whole-canopy system. Moreover, work conducted by Gadoury *et al*. (2000)^41^ on cv. ‘Concord’ over four years indicates that a PM incidence varying from 5.4 to 10 infected leaves per shoot caused no change in vine yield and a reduction in the total number of nodes with periderm from 8 to 19% in the unsprayed vines. Notably, such a reduction never translated into a reduction of yield the following year. Conversely, PM had a differential impact on must composition of PS- and FS-SG, which shared a similar technological maturity (TSS, TA and pH values reported in Table 5) but the latter had more than a double count of anthocyanins. While similar TSS and total sugar per vine nicely accord with similar seasonal NCER, the spectacular reduction in berry pigmentation recorded in PS-SG seems connected to infection directly present on clusters. Pioneer work conducted on field-grown cv. ‘Sangiovese’ in the Chianti Classico area by Amati *et al*.^42^ and Piermattei *et al*.^43^ showed that anthocyanin counts in grape skin and wines were more than halved in infected vs. healthy bunches. The latter also reported an increase in caftaric acid and in the phytoalexin *trans*-resveratrol, both of which are involved in plant resistance mechanisms. Unfortunately, neither team of authors reports leaf function, yield and other must composition parameters. A more detailed study was performed for three years on cv. Cabernet Sauvignon by infecting healthy clusters with an increasing known fraction (up to 50%) of PM diseased berries^44^. It showed that while sugar concentration increased primarily due to a reduced volume of diseased berries, promoted as well by faster transpiration loss through the cracked berry skin, the anthocyanin count decreased at the notable rate of 0.7–0.9 per percentage of mildewed berries added to the intact harvest. More recently, a detailed study on leaf transcriptome in Chinese wild grapes (*V*. *pseudoreticulata* Baihe 35-1) infected with PM shows that secondary metabolism, such as flavonoid biosynthesis, was also stimulated in response to PM^45^. In this connection, genes responsible for the biosynthesis of lignin, phytoalexins and anthocyanins were upregulated.

Overall, our data indicate that the new fungi-resistant Accession 72–096 may be a viable choice in the vineyard and winery for the features noted *supra*. On a more methodological basis, our work questions the accuracy by which single-leaf, gas-exchange readings performed on leaves where portions of green tissues coexist with infected tissues are able to represent actual whole canopy response. In our case, end of season single-leaf readings appeared to overestimate the limitation of leaf function due to PM sand DM infections in PS-SG vines, which did not show significant differences vis-à-vis 72–096 under whole-canopy assessment, a result likely due to counteracting effects linked to a compensation mechanism by healthy tissues. Our data suggest too that PM infection can lead to a decoupling in sugar-color accumulation patterns.

## Materials and Methods

### Plant material and treatment layout

The trial was conducted in 2015 at Piacenza (45°02′N, 9°43′E), Italy, on four-year-old spur-pruned cv. ‘Sangiovese’ (clone R10) and accession 72–096 (cvs. ‘Sangiovese’ x ‘Bianca’) vines both grafted to SO4 and grown outdoors in 40 L pots. According to the description provided in VCR (2015), this accession carries resistance genes for both DM and PM. Note that two sprays are recommended early in the season to safeguard resistance durability and prevent outbreaks of severe late-season infections. This protocol is aimed at reducing inoculum source and has the dual advantage of lowering disease pressure and the risk of mutations in the pathogen population: the greater this risk is, the greater the likelihood of a breach in plant resistance.

Eighteen vines were arranged along a single, vertically shoot-positioned cordon, 35° NE-SW oriented and hedgerow-trained row. Each vine had ∼1 m long fruiting cane of 8–9 nodes that was raised 90 cm from the ground with three pairs of top catch wires for a canopy wall extending about 1.3 m above the main wire. The pots were filled with a mixture of sand, loam and clay (65%, 20% and 15% by volume, respectively) and kept well-watered throughout the season. Pots were pale-green colored to limit radiation-induced overheating, and each vine was fertilized twice, one week before and two weeks after bud-break, with 4 g of Greenplant 15 (N) + 5 (P_2_O_5_) + 25 (K_2_O) + 2 (MgO) + micro (Green Has Italia, Cuneo, Italy).

The vines were assigned in winter to fully sprayed ‘Sangiovese’ (FS-SG), partially sprayed ‘Sangiovese’ (PS-SG) and partially sprayed Accession 72–096 (PS-Access). Twelve vines, six of PS-SG and PS-Access each, were randomly assigned the same spray number on the same date; the six FS-SG vines were used as control to reproduce standard practice. Spray dates were based in risk evaluation for PM and DM provided by the Decision Support System vite.net®: the treatments were applied on 14 and 28 April with a mixture of the protectant and systemic fungicides Zolvis 80 + Karatane + Kasco MZ; SG-FS received 12 sprays. Shoots in all treatments were trimmed on 21 June (DOY 172) in order to limit vegetation growth within 20–30 cm above the top foliage wires; no further trimming was performed.

### Vine growth, yield components and grape composition

Total length and the leaf area of 10 shoots of PS-SG and PS-Access were determined on extra vines on 14 May (DOY 134) to estimate total leaf area per vine before whole-vine gas exchange measurements started. Leaf area was calculated by measuring the surface of each main and lateral leaf blade with an LI-3000A leaf-area meter (LI-COR Biosciences, Lincoln, NE, USA). Shoot length (y, cm) ranged between 25 and 150 cm and was correlated to the corresponding leaf area (x, cm^2^); the resulting regression equations were y = 14.894x, R^2^ = 0.88 for SG-PS and y = 12.605x, R^2^ = 0.83 for 72–096. Total lengths were also taken on all shoots of the PS-SG and PS-Access vines, and vine leaf area was calculated from the above equations and concurrently performed shoot counts. The same procedure was repeated on 21 June (DOY 172) as an additional estimate of total leaf area per vine at a more advanced date during the growing season. Soon after the 1 September harvest, all vines, including those of SG-FS, were defoliated and the surface of each blade was processed with the leaf-area meter, keeping the contribution of main and lateral leaves separate.

The onset of veraison was estimated on 14 July (DOY 195) by random sampling of 100 berries per treatment, taking care that exterior and interior cluster sides and their top, mid and bottom positions were represented. Each berry was classified by sight and touch as green-hard, green-firm, green-soft, translucent, slightly colored-soft, colored-soft, and then processed for equatorial diameter, fresh mass and total soluble solids concentration (TSS).

Each vine was individually picked on 1 September (DOY 244) and all clusters were counted and weighed. Yield sub-samples representing clusters located in basal, median and apical sections of the fruiting cane were taken: two samples of 100 and 20 berries for each were weighed and stored at −20 °C for subsequent analyses. Cluster compactness was expressed as the ratio of total berry fresh mass to rachis plus main wing length ratio^46^. All the remaining crop from each vine sub-sample was crushed, must total soluble solids concentration (°Brix) determined and titratable acidity (TA) measured by titration with 0.1 N NaOH to a pH 8.2 end point and expressed as g/L of tartaric acid equivalents.

### HPLC analyses

All solvents were of HPLC quality and all chemicals of analytical grade (>99%). Water Milli-Q quality, acetonitrile and methanol were supplied by VWR (Radnor, PA, USA). Formic acid, gallic acid, syringic acid, (+)-catechin, (−)-epicatechin, vanillin and HCl were purchased from Sigma-Aldrich (St. Louis, MO, USA). The following commercial standards from Extrasynthese (Genay, France) were used: protocatechuic acid, procyanidine B2, resveratrol, myricetin, myricetin 3-O-glucoside, quercetin 3-O-glucuronide, quercetin 3-O-glucoside, kaempferol 3-O-glucoside, delphinidin 3,5-O-diglucoside, cyanidin 3,5-O-diglucoside, peonidin 3,5-O-diglucoside, malvidin 3,5-O-diglucoside, delphinidin 3-O-glucoside, cyanidin 3-O-glucoside, petunidin 3-O-glucoside, peonidin 3-O-glucoside and malvidin 3-O-glucoside.

The berry samples were manually and carefully peeled, and the resulting skins and seeds were immediately freeze-dried. Phenolic compounds were extracted from skins after Downey and Rochfort^47^: 0.100 g of freeze-dried skin were extracted in 1.0 mL of 50% (v/v) methanol in water for 15 min with sonication. The phenolic compounds of seeds were extracted after Nicoletti *et al*.^48^ with minor modification: 0.200 g of freeze-dried seeds were extracted in 25 mL of methanol/ethanol (8:2, v/v) by sonication for 15 min, shaken for 12 h in a round-bottom flask at room temperature, centrifuged at 10000 × g at 4 °C and extracted twice for 2 h using 5 mL of fresh extraction solvent; supernatants were collected and concentrated in a rotavapor in a 35 °C water bath, the residue was recovered in 1.5 mL of 50% (v/v) methanol in water. The seed and skin extracts were centrifuged for 5 min at 10000 × g at 4 °C, filtered through a 0.22 µm polypropylene syringe for HPLC analysis and transferred to HPLC auto-sampler vials.

The chromatographic method was developed using an Agilent 1260 Infinity Quaternary LC (Agilent Technology, Santa Clara, CA, USA). It consisted of a G1311B/C quaternary pump with inline degassing unit, G1329B autosampler, G1330B thermostat, G1316B thermostat column compartment and a G4212B diode array detector fitted with a 10 mm path, 1 µL volume Max-Light cartridge flow cell. The instrument was controlled using Agilent Chemstation software version A.01.05. Separation was achieved on a reverse-phase C-18 Synergi Hydro RP 80 A, 250 × 4.6 mm, 4 µm (Phenomenex, Torrance, CA, USA). The solvents used were 5% (v/v) formic acid (solvent A) and acetonitrile (solvent B). The flow rate was 0.5 mL/min, with a linear gradient profile consisting of solvent A with the following proportions (v/v) of solvent B: 0–10 min, 2–10% B; 10–25 min, 10–12% B; 25–35 min, 12–30% B; 35–43 min, 30% B; 43–48 min, 30–40% B; 48–52 min, 40–50% B; 52–55 min, 50–60% B; 55–58 min, 60–98% B; 58–63 min, 98% B; 63–66 min, 98–2% B; 66–72 min 98% B. Column temperature was kept at 40 ± 0.1 °C. Five microliters of sample extract were injected. The elution was monitored at 200–700 nm with detection by UV-Vis absorption with DAD scanning between 280, 320, 370 and 520 nm. Phenolic compounds were identified using authentic standards and by comparing retention times. Quantification was based on peak areas and performed by external calibration with standards. Petunidin 3,5-O-diglucoside, acylated and coumarated anthocyanins were identified by comparison to data available in literature. All anthocyanins were expressed as malvidin 3-O-glucoside.

### Disease assessment

Inspections were conducted on 28 June (BBCH 77 – berries beginning to touch) and 30 August (BBCH 89 – berries ripe for harvest) on both leaves and clusters^49^. Disease incidence and severity were estimated: the former as the proportion of diseased leaves on a sample of 20 random leaves per pot (120 leaves per treatment) and the latter as the proportion of diseased bunches out of total bunches per plant. Severity was assessed on the 20 random leaves as the percentage of leaf area affected by DM (caused by *Plasmopara viticola* (Berk. & Curt., Berl. & de Toni), PM (*Erysiphe necator* Schw.), and the grape erineum mite *Colomerus vitis* Pagenestecher; no other disease symptoms or pest damage were observed. Severity was also assessed for all clusters on the six vines per treatment by visually estimating each leaf and cluster using EPPO descriptors and assigning them to one of the following classes: 0%, no symptoms, <5% of leaf (or cluster) area affected, 5 to 25%, 25 to 50%, and >50% (OEPP, 2002). Formulas used for calculation of disease incidence and severity were the following;

1 $${\rm{Disease}}\,{\rm{incidence}}=\frac{{\rm{d}}}{({\rm{h}}+{\rm{d}})}\ast 100$$

where *h* is the number of the healthy leaves (or bunches) and *d* is the number of the diseased leaves (or bunches);

2 $${\rm{Disease}}\,{\rm{severity}}=\frac{{\sum }_{(i=1)}^{n}({\rm{x}}\ast {\rm{s}})}{({\rm{h}}+{\rm{d}})}$$

where n is the number of severity classes; *x* is the number of leaves (or bunches) per class; *s* is the average of severity range per each class; *h* is the number of the healthy leaves (or bunches) and *d* is the number of the diseased leaves (or bunches).

### Single-leaf and whole-canopy gas exchange

Leaf net assimilation (A), transpiration (E) and stomatal conductance (g_s_) rates of well-exposed, mature primary and lateral leaves were measured on 18 May (DOY 138), prior to whole-canopy system setup, using a CIRAS-2 portable photosynthesis system (PP Systems, Amesbury, MA, USA). Two primary leaves inserted on node 4 and 8 of each of two shoots per vine were sampled at each measurement. The two sampled shoots were usually the first basal and the apical on each cane.

Light response curves and leaf dark respiration response curves to leaf temperature were measured on 5 fully expanded leaves per treatment on 22 June (DOY 173). Full sun-exposed leaves were placed in the leaf chamber and light intensity progressively decreased until darkness by adjusting the LED lamp of the CIRAS-2; leaves were left to reach steady state before recording. Once the dark condition was reached, temperature was increased at ∼2 °C steps up to 43 °C; records were logged at each step after leaf equilibration.

Soon after the 1 September harvest (DOY 244), three primary leaves inserted on the basal, median and apical portions on each of two shoots per vine were sampled for gas exchange using the same equipment. The first or second fully expanded basal leaf of the lateral that had developed underneath the trimming cut was also measured. All leaves showing signs olf PM and DM were rated on an arbitrary severity scale ranging from 0 (symptomless) to 5 (very heavy).

Readings were performed from 10:00–12:00 under constant saturating light (≅1500 μmol m^−2^⋅s^−1^) imposed with an additional external lamp mounted on top of the leaf chamber. Measurements were taken at ambient relative humidity, and the flow fed to the broad-leaf chamber (4.5 cm^2^ window size) was 300 mL min^−1^. To ensure stability of the inlet reference CO_2_ concentration [CO_2_], a mini CO_2_ cartridge was used to provide automatic control of inlet (CO_2_) at 380 mmol L^−1^.

Whole-canopy net CO_2_ exchange rate (NCER) measurements were taken using the multi-chamber system after Poni *et al*.^36^. It features alternating current, centrifugal blowers (Vorticent C25/2 M Vortice, Milan, Italy) delivering a maximum air flow of 950 m^3^ h^−1^, flexible plastic polyethylene chambers allowing 88% light transmission, 6% diffuse light enrichment and no alteration of the light spectrum, a CIRAS-EGM4 single channel absolute CO_2_ infrared gas analyzer (PP-Systems, Amesbury, MA), and a CR1000 data logger wired to an AM16/32B Multiplexer (Campbell Sci., Shepshed, UK). Switching of air sampling from one chamber to another was at programmed time intervals using a set of solenoid valves; the air-flow rate to each chamber was controlled by a butterfly valve and measured with a Testo 510 digital manometer (Farnell, Lainate, Italy) after the flow restriction method described in Osborne^50^.

Twelve chambers for each PS-SG and PS-Access vine were set up on 28 May (DOY 148, post-bloom) and operated 24 hours a day until 21 June (DOY 172) (Fig. 1). Chambers were temporarily dismantled the next day and vines left un-chambered for 15 days in order to allow disease assessment, shoot trimming, access for single-leaf gas exchange readings, and system repair and maintenance. Chambers were then reassembled on 7 July (DOY 188, pre-veraison) and left in place until final pre-harvest disassembly on 31 August (DOY 243).

The air flow rate fed to the chambers was progressively adjusted according to the increasing leaf area in the chambers. It varied from 10.1 L s^−1^ between DOY 147–157 and was then raised to 14.4 L s^−1^ on DOY 158 and maintained constant during the remainder of the season. Ambient (inlet) air temperature and the air temperature at each chamber outlet were measured by shielded 1/0.2 mm diameter PFA –Teflon insulated type-T thermocouples (Omega Eng. INC, Stamford, Connecticut); direct and diffuse radiation were measured with a BF2 sunshine sensor (Delta-T Devices Ltd, Cambridge, UK) placed horizontally on top of a support stake next to the chambers enclosing the canopies. Canopy NCER (µmol CO_2_/s) was calculated from flow rates and CO_2_ differentials after Long and Hallgren^51^.

### Statistical treatmen

One-way analysis of variance (ANOVA) was carried out and, in case of significance of F test, mean separation was performed by the Student-Newman-Keuls test at *P* < 0.05 and 0.01. BBCH readings were transformed into root squared values prior to analysis. Table-Curve 2D (Systat Software Inc. London, UK) was use to run polynomial regressions. Degree of variation around means is given as standard error (SE). Due to the high number of measured variables, a Principal Component Analysis (PCA) was also carried out using the XLSTAT statistical package (Addinsoft, New York, USA). Observations were single vine data for the three biotypes, whereas 14 variables were analysed to include all those reported in Table 4 and, in addition, TSS, TA, pH and total anthocyanins. The chosen PCA was a Pearson correlation matrix, number of filter factors was set at 5 and the final data visualization was in the form of a distance bi-plot.

Disease incidence and severity data were transformed using the arcsin function to make variances homogeneous. The arcsin values were then used in a one-way analysis of variance (ANOVA) to test the effects of treatments, assessment time and their interaction. The Fisher Protected Least Significant Difference (LSD) Test was used for mean comparison at P = 0.05.

## Electronic supplementary material

Supplementary Information
