# Supplementary material for: Canopy physiology, vine performance and host-pathogen interaction in a fungi resistant cv. Sangiovese x Bianca accession vs. a susceptible clone
Source: Sci Rep. 2017 Jul 20;7:6092. doi: 10.1038/s41598-017-05530-7 (PMC5519749; doi:10.1038/s41598-017-05530-7)
Supplement: Supplementary file 1 — Supplementary Information [file 41598_2017_5530_MOESM1_ESM.doc]

Supplementary information for manuscript titled: **Canopy physiology, vine performance and host-pathogen interaction in a fungi resistant cv. Sangiovese x Bianca accession vs. a susceptible clone.**

By:

S. Poni^1^, G. Chiari^1^, T. Caffi^1^, F. Bove^1^, S. Tombesi^1^, A. Moncalvo^1^, M. Gatti^1^.


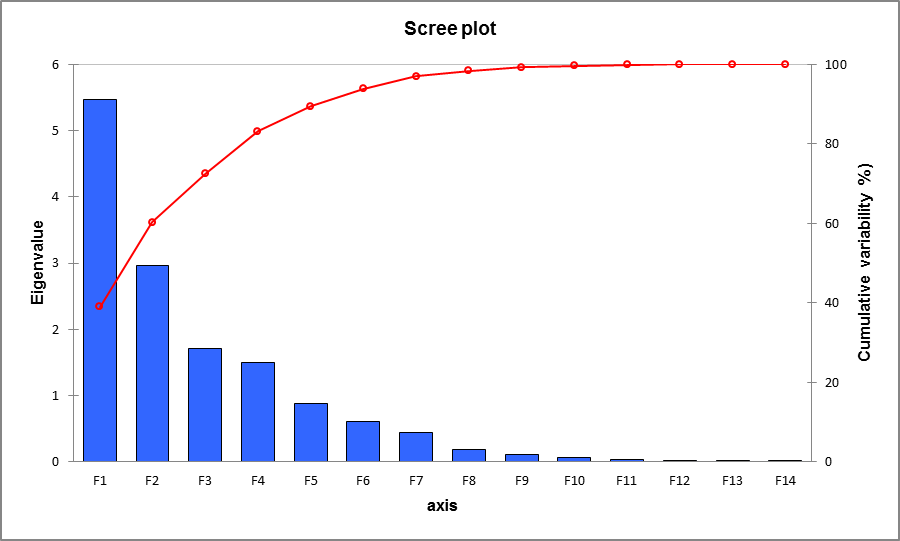


Supplementary Figure 1. Scree plot displaying the eigenvalues associated with a component or factor in descending order versus the number of the component or factor.

Supplementary Table 1. Correlation coefficients between variable and factors.

Values higher than 0.6 are highlighted in bold character

|  | F1 | F2 | F3 | F4 | F5 |
| --- | --- | --- | --- | --- | --- |
| Shoots/vine | 0.347 | 0.562 | -0.034 | 0.273 | **-0.649** |
| Clusters/vine | 0.533 | **0.738** | -0.304 | -0.090 | -0.070 |
| Yield/vine | **-0.615** | **0.686** | -0.266 | -0.095 | 0.153 |
| Total LA | 0.286 | 0.516 | -0.062 | **0.633** | 0.460 |
| Berry weight | **-0.864** | -0.095 | -0.297 | 0.207 | 0.053 |
| Cluster weight | **-0.938** | 0.187 | 0.093 | 0.063 | 0.087 |
| Rachis length | **-0.765** | 0.141 | 0.358 | 0.283 | -0.127 |
| Berry number | **-0.634** | 0.402 | 0.102 | -0.138 | -0.097 |
| TSS (°Brix) | **0.941** | 0.015 | -0.041 | -0.205 | 0.063 |
| TA | **0.670** | 0.374 | 0.579 | -0.037 | -0.097 |
| pH | 0.301 | -0.581 | **-0.624** | -0.191 | -0.008 |
| LA/Y | **0.717** | -0.160 | 0.187 | 0.573 | 0.234 |
| Total sugars | 0.213 | **0.797** | -0.342 | -0.365 | 0.214 |
| Anthocyanins | -0.041 | 0.067 | **0.660** | -0.567 | 0.261 |


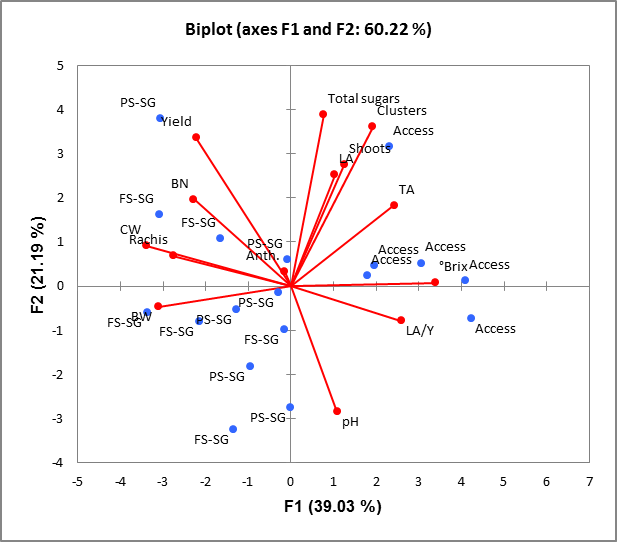

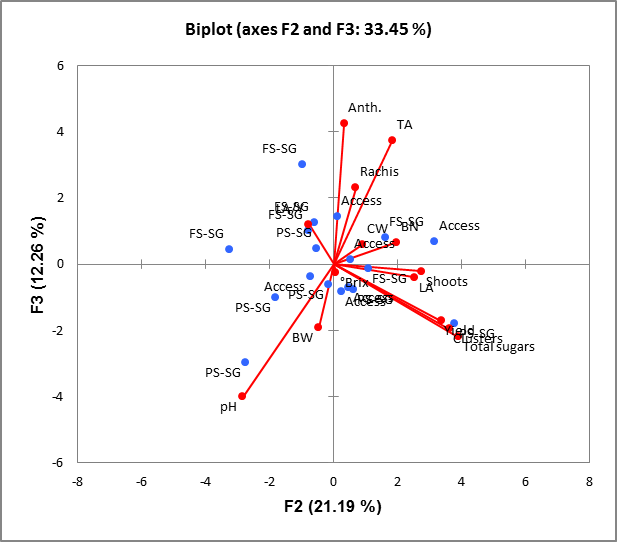


Supplementary Figure 2. Top: biplot for F1 and F2. Bottom: biplot for F2 and F3.

Blue dots identify location of each sample, red dots refer to variables.
